# Two Cases of Tracheotomy

**Published:** 1883-02-20

**Authors:** C. G. Jennings

**Affiliations:** Detroit


### TWO CASES OF TRACHEOTOMY.

By C. G. Jennings, M. D., Detroit.

Case I.—John McDonald, æt. nineteen months. During the night of November 8th he began to cough hoarse and have attacks of difficult breathing. This passed off the next day but returned the following night. He was first seen on the morning of November 10th. He then coughed croupy, was aphonic and showed slight embarrassment in respiration. Temperature 99°; pulse slightly accelerated; no exude visible in the throat; no swelling of the cervical glands. The dyspnœa again became great toward night, and by noon the next day the stenosis had so advanced, that other measures having failed to give relief, tracheotomy was decided upon. The treatment to this time had been the use of the steam atomizer with a solution of bicarbonate of sodium ten to fifteen minutes out of every half hour; the administration of two grains of quinine every three or four hours; expectorant doses of syrup of ipecac continuously, and emetic doses when the breathing was very difficult.

At 3:30 o'clock p. m. the child was in the third stage of the disease. The paroxysms had ceased, the dyspnœa was continuous and great, and cyanosis was becoming marked. Assisted by Drs. Campau and Miner, with the patient under chloroform, I opened the trachea above the thyroid isthmus, incising the cricoid cartilage and enough of the trachea to admit a small canula. There was no exudate below the opening, and the child breathed freely. The tube produced no irritation, and the little patient slept quietly for several hours after the operation. Nine o'clock p. m.—Temperature, 102°; pulse, 150; respiration; 30. He coughed a little bloody mucus.

November 12th.—Passed a very quiet night; coughed but little and no difficulty was experienced in keeping the tube clear. Temperature, 101°; pulse, 140; respiration, 36.

November 14th.—Last night he coughed up a membranous cast of the trachea one inch long. The canula was removed to wash the wound. The lower part of the wound was healed by first intention.

From this time the case went on favorably. No diphtheritic exudate appeared on the wound and no complications arose, except a very slight bronchitis on November 29th and 30th. For several days previous to this the larynx seemed free from exudate, but the permanent removal of the canula was delayed until December 3, twenty-two days after the operation, on account of collapse of the anterior wall of the trachea, which would take place at every deep inspiration. The tube could be left out for several hours if the child breathed quietly, but it would have to be replaced immediately if he began to cry. This condition, as I showed in the report* of a previous case, is quite liable in very young children to delay the permanent removal of the canula.

*New York Medical Record. Oct, 1, 1881.

At the present writing the external wound has not completely closed but it is healing rapidly. Vocalization is perfect.

CASE. 2. Lillie G., æt. 7 years and 8 months. This patient was taken ill with pharyngeal diphtheria November 10th. The exudate extended over the tonsils, pharynx, uvula and arches of the soft palate. There was considerable swelling of the cervical glands and the constitutional symptoms were quite severe. On the 15th, when the pharyngeal disease was declining, she became hoarse and aphonic, and in a few hours the shrill cough and dyspnœa indicated the extension of the exudate into the larynx. The treatment for the pharyngeal disease was continued, and the steam atomizer with sodium bicarbonate used every half hour. For five days the symptoms of larynxgeal stenosis continued with varying severity. Encouraging remissions would take place every morning, and once or twice complete relief followed the separation of large pieces of membrane. The nocturnal exacerbations were quite alarming at times, but were not sufficiently prolonged to demand operative interference. The child refused to take nourishment during this period and became quite weak and emaciated. The course of treatment pursued was the administration of two grains of quinine in syrup every four hours; expectorant, and at times emetic doses of ipecac; stimulants in moderate doses; tincture of the chloride of iron; and the use of the steam atomizer almost continuously with a solution of sodium bicarbonate,alternating with a very weak solution of sodium hydrate. Two emetic doses of turpeth mineral were given one night when the breathing was very difficult, but no relief followed. The morning of the 20th did not bring the usual remission, and early in the afternoon it was decided that tracheotomy offered the only hope of prolonging the patient's life.

The condition of the patient was quite unpromising for the operation. A thin diphtheritic membrane still covered the pharynx, uvula and arches; the glandular swelling had not entirely subsided. The dyspnœa was great, the face pale and perspiring, lips dark and finger nails blue; great depression of the epigastrium at every inspiratory effort. Pulse, 160 and very weak. The little patient was completely exhausted by her long struggle for air. The only circumstance which gave me a particle of hope for the operation was the fact that the diphtheritic process had reached its height and was rapidly declining. If we could avert the impending suffocation, and by the relief it would afford conserve the patient's strength for a few days, the larynx would throw off the obstructing membrane and a cure follow.

At 3:30 o'clock p. m., assisted by Drs. Campau and Miner, I performed a laryngotracheotomy, incising the cricoid membrane and cartilage, and two or three rings of the trachea. A teaspoonful or more of pus came through the opening and a large piece of membrane was removed with the forceps. After the trachea was cleansed, and appeared perfectly free below the opening the canula was inserted and respiration went on easily. Ten o'clock p. m.: Temperature, 102°; pulse, 140; respiration, 24.

November 21. Passed a very quiet night, sleeping most of the time. Is much stronger. Temperature, 90°; pulse, 100; respiration, 24; takes some nourishment.

November 23 Removed the canula from the wound this morning. A large patch of diphtheritic exudate extends downwards from the lower end of the wound. She coughed through the wound almost a complete membranous cast of about three inches of the trachea.

November 25. The slight extension of the diphtheria mentioned in last note has quite prostrated the patient. She again refuses nourishment. Pulse 150, very weak; temperature, 100. The exudate on wound has been kept covered with powdered iodoform and it now has disappeared. The larynx also is clearing up.

November 26. Found the larynx perfectly free this morning. Left the canula out and dressed the wound with a large pad of absorbent cotton and iodoform vaseline. Liquid food comes out through the wound in attempts to swallow. Temperature, normal; pulse, 160. Patient is very weak. Ordered peptonized beef, elixir of calisaya. iron and strychnia, and sweet cream and brandy *ad libitum*.

For two days this state of alarming prostration continued. Then her appetite returned. She rapidly gained strength, and in a short time was convalescent. At this time the wound is not entirely healed, but is closing rapidly. She is still aphonic.

These two cases are of considerable interest, since recovery took place in both, when there was apparently but little to be hoped, on account of the tender age of the patient in the first case, and the asthenia in the second.

The operation on children under the age of two years, has not been very successful. With some operators it has been so uniformly fatal, that they consider this age a contra indication to the operation. The most complete statistics of tracheotomy in the United States are published by Dr. Wm. M. Martin, of Mobile. in Gaillard's Medical Journal for January, 1880. He gives a table of 32 operations on infants under two years of age, with five cures and 27 deaths, a proportion of one cure to 6 2-5 cases. Dr. Geo. F. Shardy, in a recent number of the New York Medical Record, publishes a successful case at the age of 11 months. This would make the published statistics, including the writer's case, show seven cures in 24 operations.

I have considered these cases to be diphtheritic croup. Although in the first case there were no constitutional symptoms manifest during the whole course of the disease, the epidemic in the neighborhood and a clear history of long exposure, leave no doubt in my mind that the disease was diphtheritic in its origin. I attended a sister of the child, who had diphtheria, a week or two before his attack, and he remained in the room with her during her entire illness. Four other children, who were sent away, escaped.—*Detroit Clinic.*

